# A Kalman Filtering and Nonlinear Penalty Regression Approach for Noninvasive Anemia Detection with Palpebral Conjunctiva Images

**DOI:** 10.1155/2017/9580385

**Published:** 2017-07-30

**Authors:** Yi-Ming Chen, Shaou-Gang Miaou

**Affiliations:** ^1^Acoustic Science and Technology Laboratory, College of Underwater Acoustic Engineering, Harbin Engineering University, Harbin, China; ^2^Department of Electronic Engineering, Chung Yuan Christian University, Taoyuan City, Taiwan

## Abstract

Noninvasive medical procedures are usually preferable to their invasive counterparts in the medical community. Anemia examining through the palpebral conjunctiva is a convenient noninvasive procedure. The procedure can be automated to reduce the medical cost. We propose an anemia examining approach by using a Kalman filter (KF) and a regression method. The traditional KF is often used in time-dependent applications. Here, we modified the traditional KF for the time-independent data in medical applications. We simply compute the mean value of the red component of the palpebral conjunctiva image as our recognition feature and use a penalty regression algorithm to find a nonlinear curve that best fits the data of feature values and the corresponding levels of hemoglobin (Hb) concentration. To evaluate the proposed approach and several relevant approaches, we propose a risk evaluation scheme, where the entire Hb spectrum is divided into high-risk, low-risk, and doubtful intervals for anemia. The doubtful interval contains the Hb threshold, say 11 g/dL, separating anemia and nonanemia. A suspect sample is the sample falling in the doubtful interval. For the anemia screening purpose, we would like to have as less suspect samples as possible. The experimental results show that the modified KF reduces the number of suspect samples significantly for all the approaches considered here.

## 1. Introduction

According to WHO [1], anemia is a prevalent health problem affecting an estimate of 2 billion people in many parts of the world, especially where dietary iron deficiency, malaria, and hookworm infections are common. Blood test is the most common way for the anemia assessment based on the level of hemoglobin (Hb) concentration. A normal blood test procedure needs drawing blood from a vein or a finger stick, which is all right for most people but not acceptable for those who suffer from blood phobia and fainting during acupuncture. In addition, phlebotomists, laboratory practitioners, and nurses are charged with drawing a patient's blood, and training those people to have enough professional skills is also the hidden cost that cannot be overlooked. Besides, the entire procedure of blood test may take more than an hour, which limits the number of patients that could be examined in a day and makes the large-scale anemia screening difficult. In summary, the traditional way of drawing blood for anemia assessment is time consuming and costly and not appropriate for everyone. Therefore, an efficient way to screen out anemia patients for essentially anyone is desirable.

**Figure 1:**
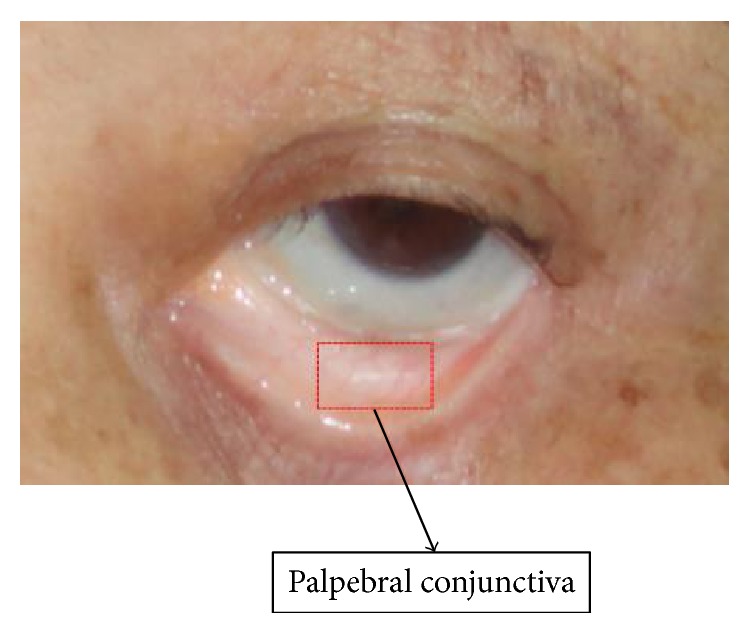
The palpebral conjunctiva part of an eye.

**Figure 2:**
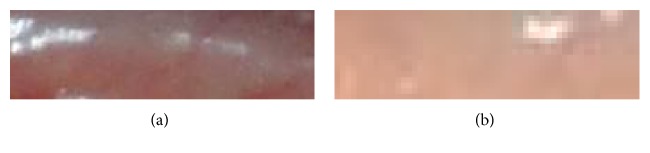
Cropped palpebral conjunctiva samples. (a) Nonanemic sample; (b) anemic sample.

In view of the considerations above, a great number of studies on noninvasive approaches for Hb estimate as well as anemia test have been conducted in the recent years [2–14]. One popular solution is to crop a small image within the digital photo of the palpebral conjunctiva area (Figure 1). Physicians can diagnose a patient's anemia condition based on the pale degree observed from the image, such as the nonanemic sample in Figure 2(a) and the anemic sample in Figure 2(b). Sometimes, image samples taken in poor condition are not as good as those presented in Figure 2. The quality of the image is obviously the key to the success of this kind of solution. Hence, some researchers study how to acquire palpebral conjunctiva images more reliably, and this is usually accomplished by reducing its dependency on ambient light condition. These include photo plethysmography [4] and reflectance spectroscopy [5], as well as a head-mounted device with LED array inside to capture conjunctiva images [12]. However, they are not popular or affordable, especially in the rural areas of developing countries where medical resources are often limited. Thus, we get data acquisition back on track by using a more popular and affordable device such as a commercially available digital camera. At the same time, we need to deal with the biggest potential problem of this kind of approach, that is, the threat from noisy sources, including various ambient light conditions and camera settings.

The noise effect results in the interference of extracting color feature and makes the anemia assessment more susceptible to error. Therefore, a preprocessing step before color extraction is desirable. Few preprocessing efforts have been made specifically for noninvasive anemia examining. In [13, 14], a preprocessing algorithm based on k-mean clustering is tailored to remove the bright reflection spot in the image. However, this is just one type of special noises due to camera setting and/or ambient light condition and the k-mean clustering approach is not general enough to deal with all possible noise types. In fact, applying k-mean clustering to the image with no bright reflection may even introduce unwanted man-made noise. Therefore, a more general solution with low probability of adverse effect is considered in this paper.

We treat the bright reflection or any other noise as simply a measurement error. Apparently, if a preprocessing process was not able to eliminate all the noise in the first step, the corresponding measurement error would add difficulty to the next step—feature extraction. Being aware of it, some noninvasive methods for anemia assessment attempt to obtain image samples as clean as possible in the very beginning with various rather sophisticated equipment [4–7]. With no perfect preprocessing method or such special equipment, our prime work is therefore aimed at reducing the overall measurement error. The idea is connected to one of the most famous algorithms—Kalman filter (KF). Although it is commonly used in the problem of position and orientation tracking, its main function stays unchanged, that is, to optimize each data measurement and estimation. Therefore, we modify the traditional KF such that it can be applied to our current problem. In fact, there have been examples of applying the KF for medical fields, such as those in [15, 16], where Kalman filters were applied to medical image reconstruction and tracking blood vessel, respectively. Another application is in signal filtering, which is more direct and easily understood. Foussier et al. in [17] used KF to reduce the measurement error of cardiorespiratory signal, resulting in improved signal quality for further processing. In spite of different applications above, they share a common characteristic, that is, Kalman filtering is often used to deal with the data with time-dependent nature. In this paper, we apply it to the time-independent data derived from an original medical signal, which is unique to the best of our knowledge. Specifically, a feature sample extracted from a palpebral conjunctiva image is regarded as a dependent variable, while the corresponding Hb level is treated as an independent variable (equivalent to time variable in traditional KF). As discussed above, each computed feature has somehow inherited measurement error from the original data. Therefore, applying KF in such setting is plausible.

Regression analysis is widely used for prediction and forecasting, where its use has substantial overlap with the field of machine learning. Regression analysis is also used to understand which among the independent variables are related to the dependent variable and to explore the forms of these relationships. In restricted circumstances, regression analysis can be used to infer causal relationships between the independent and dependent variables. However, this can lead to illusions or false relationships [18], such as overfitting. In overfitting, a statistical model describes random error or noise instead of the underlying relationship. Overfitting occurs when a model is excessively complex, such as having too many parameters relative to the number of observations. An overfitting model usually has poor predictive performance, as it overreacts to minor fluctuations in training data [19]. For medical applications, physicians often do not know how large a sample measurement error could be. It is expected that the fitting relationship from medical data will appear to perform slightly worse on a new data set than on the data set used for fitting and prediction, which would cause a serious problem for both patients and physicians. Therefore, we add penalty consideration in the regression analysis to avoid the overfitting problem. Penalty methods are a class of algorithms for solving constrained optimization problems [20].

In this paper, we propose a combining algorithm based on a modified Kalman filter and the nonlinear penalty regression for anemia assessment. We simply compute the mean value of the red (R) component of conjunctiva images and consider it as the only recognition feature to simplify the proposed system. Then, given the data of these mean values, we use the combining algorithm to fit a nonlinear curve that presents the relationship between the feature value and the Hb level. With the fitting curve, we then propose a three-level evaluation scheme to determine the anemia status of a given subject as well as the performance of the proposed approach.

## 2. Method

**Figure 3:**
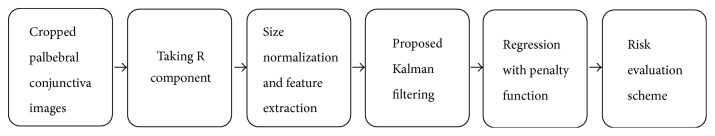
A flow chart of the proposed approach.

A flow chart of the proposed approach is shown in Figure 3. The original data at hand consist of *N* palpebral conjunctiva images of unequal sizes, along with *N* corresponding Hb levels, taken from *N* subjects, respectively. Firstly, we consider only the R component of each cropped palpebral conjunctiva image. Each image is then normalized to the same size. Next, for each normalized image, we simply take the mean value of R component pixels as the only one recognition feature in our approach to simplify the proposed system. Since we perform 10-fold cross validation in this work, about 90% and 10% of the images and the corresponding features values shall be used in training and testing, respectively. Given the training images and the corresponding Hb levels, we propose and perform a modified version of Kalman filtering. In the next step, regression with penalty function is used to find the relationship between feature values and Hb levels. Finally, a three-level evaluation scheme called risk evaluation scheme (RES) is proposed for the testing images to signify the subject's anemia status and evaluate the performance of the proposed approach as well.

### 2.1. Traditional Kalman Filter

The Kalman filter (KF) was proposed as a novel approach for the state estimation of dynamic linear state system in [21], and it has been widely implemented in various applications [22–29].

The KF is analogous to the Markov model, where it needs an estimate state from the previous time instant and the current measurement to calculate the estimate for the current state. The KF is usually conceptualized as two distinct phases, time update phase ((1) and (2)) and measurement update phase ((3), (4), and (5)) [30].

(1) $$\begin{array}{c} x_{\left.k\right|k-1}=F_{k}x_{\left.k-1\right|k-1}+B_{k-1}u_{k-1}, \end{array}$$

(2) $$\begin{array}{c} P_{\left.k\right|k-1}=F_{k}P_{\left.k-1\right|k-1}F_{k}^{T}+Q_{k}, \end{array}$$

(3) $$\begin{array}{c} K_{k}=\frac{P_{\left.k\right|k-1}H_{k}^{T}}{H_{k}P_{\left.k\right|k-1}H_{k}^{T}+R_{k}}, \end{array}$$

(4) $$\begin{array}{c} x_{\left.k\right|k}=x_{\left.k\right|k-1}+K_{k}\left(z_{k}-H_{k}x_{\left.k\right|k-1}\right), \end{array}$$

(5) $$\begin{array}{c} P_{\left.k\right|k}=\left(I-K_{k}H_{k}\right)F_{k}P_{\left.k-1\right|k-1}F_{k}^{T}+\left(I-K_{k}H_{k}\right)Q_{k}. \end{array}$$

Here, the KF model computes the estimate state *x*_*k*∣*k*_ at time *k* which is evolved from the previous estimate state *x*_*k*−1∣*k*−1_; the *F*_*k*_ is the state transition model which is applied to the previous state; the *B*_*k*_ is the control-input model which is applied to the control vector *u*_*k*_; the *P*_*k*∣*k*_ is the estimate of error covariance matrix; the *K*_*k*_ is Kalman gain; *z*_*k*_ is the observation value at the current time *k*. In practice, the process noise covariance matrix *Q* (must be positive semidefinite in theory) and the measurement noise covariance matrix *R* (must be positive definite in theory) might change with respect to time change or measurement. They are assumed to be independent (of each other), white, and normally distributed.

The time update phase is responsible for projecting forward the current state (1) and error covariance estimates (2) to obtain the first predicted estimates *x*_*k*∣*k*−1_ for the next time step. The aim of this step is to minimize the covariance of the estimation error, which represents a degree of uncertainty of the estimation [30]. The measurement update phase is responsible for updating the Kalman gain in (3), state estimate in (4), and estimate covariance in (5). In (4), *x*_*k*∣*k*−1_ is updated to the final estimate state *x*_*k*∣*k*_ at time *k*. Equation (5) for the updated estimate of covariance matrix above is only valid for the optimal Kalman gain.

In most cases, Kalman filtering is wildly applicable to the time-dependent field of navigation, radar, computer vision, and so forth, and very few works have applied it to medical data processing. In the next section, we attempt to modify the traditional KF so that it can be connected to the medical data processing task of interest.

### 2.2. A Proposed Modified Kalman Filter

The original images have various sizes, and they are normalized (enlarged) to a fixed size to avoid possible loss of useful information. Then, we compute the mean value of the R component in the RGB color space and use it as a recognition feature for anemia assessment, because the reddish and pale conjunctiva may correspond to two opposite extremes of Hb levels.

However, when the variance or standard deviation of feature values is large (due to measurement errors in data acquisition and individual differences in physiology), it is more difficult to figure out the exact relationship between the feature values and the Hb levels. Thus, we use Kalman filtering to make the samples closer to each other so that it will be easier for us to find the fitting curve representing the relationship between these two quantities. Kalman filtering is commonly used in time-dependent cases, as we discussed earlier. We map our time-independent feature Hb problem into a target position-tracking problem, which is a well-known time-dependent problem. In the tracking problem, time is the independent variable and the target position is the dependent variable with some measurement error or uncertainty. The KF is used to reduce the uncertainty. In our problem, the Hb level is regarded as an independent variable and the feature is considered to be a dependent variable, which also suffers from measurement error due to camera setting or ambient light condition. With this mapping, the same principle of KF can be applied to our problem after a proper reformulation of the original KF setting.

Firstly, we sort the measurement data *z*_original_(*k*) by Hb in an ascending order. We assume that there are enough amounts of measurement data for analysis. We also assume that the interval defined by any pair of two adjacent and sorted Hb values has small enough differences, so that we have essentially the same interval length. Furthermore, one Hb level with finite precision may correspond to multiple feature values in practice. To simplify the problem, we take the average of these feature values in this case and convert a possibly one-to-many mapping function *z*_original_(*k*) to a one-to-one counterpart *z*(*k*). Now, *k* is no longer limited to a time step; instead, it can be interpreted as a discrete number of cases considered in either ascending or descending order of Hb. We are about ready to apply Kalman filtering to our problem. For the efficient implementation of KF, we simplify the original KF by treating some complicated parameters in KF to be constants and avoiding the required iteration of their estimation as follows.

We firstly assume that the first data in the array *z*(*k*) is correct, where we have *P*(1) = 0. And there is no control vector in our case, so *u*_*k*_ is set to be 0. For the one-dimensional data in our case, let *H* = 1 (just a scaler, not a matrix anymore). We also consider the system to be stable and set *F* = 1. Extensive researches have been done to estimate noise covariance matrices *Q*_*k*_ and *R*_*k*_ from the data, such as [31–37]. A practical implementation of getting a good estimate of these covariance matrices is still difficult. Furthermore, KF is sensitive to errors in *Q*_*k*_ and *R*_*k*_, and its output can be unacceptable if errors are large. Therefore, we usually assume they are small constants within a certain range, say *Q*_*k*_ = *Q* = 1 × 10^−3^ ~ 1 × 10^−2^ and *R*_*k*_ = *R* = 1 × 10^−3^ ~ 1 × 10^−2^. Thus, after a simple derivation, the mathematical formulation of the original KF is reduced to that shown next:

(6) $$\begin{array}{c} K\left(k\right)=\frac{P\left(k-1\right)+Q}{P\left(k-1\right)+Q+R}, \end{array}$$

(7) $$\begin{array}{c} x\left(k\right)=K\left(k\right)\cdot z\left(k\right)+\left(1-K\left(k\right)\right)\cdot x\left(k-1\right), \end{array}$$

(8) $$\begin{array}{c} P\left(k\right)=\left(1-K\left(k\right)\right)\cdot P\left(k-1\right)-2\cdot Q\cdot K\left(k\right)+2\cdot Q, \end{array}$$

where *K*(*k*) is the Kalman gain. Note that (6), (7), and (8) give a simplified version of Kalman filtering according to our current research work. The simplification provides a useful guide for those who want to implement the filtering directly with their own programming languages and techniques. One handy alternative is to use the existing MATLAB function “kalman” with a proper parameter setting. In our implementation, we use the statement [output] = kalman(system_input, *Q*, *R*), where system_input is the structure array containing the input signal and the parameters such as *H* and *F*, and *Q* and *R* correspond to *Q*_*k*_ (or *Q* here) and *R*_*k*_ (or *R* here), respectively.

With the modified KF proposed above, a more condensed data *x*(*k*) than *z*(*k*) can be obtained and then used in the following step.

### 2.3. Penalty Regression

The anemia examining can be considered as a linear regression problem, since the pale degree of the palpebral conjunctiva is commonly regarded as an obviously visible feature reflecting Hb level. For example, the conjunctiva looks paler when Hb level tends to be low. However, no study has ever shown that the problem considered here can be modeled perfectly by a linear expression between the pale degree and the Hb level. Naturally, we cannot exclude the development of a nonlinear expression for our problem.

Since we take the mean value in R component as the feature, we formulate the anemia-examining problem as a predicting problem based on a fitting curve with an *n*th order nonlinear polynomial function:

(9) $$\begin{array}{c} f\left(h_{k},a_{i}\right)=\overset{n}{\underset{i=0}{\sum }}a_{i}\cdot h_{k}^{i}, \end{array}$$

where *h*_*k*_ represents *k*th subject's Hb level in an ascending order after applying the proposed Kalman filtering and *f*(*h*_*k*_, *a*_*i*_) is the corresponding function whose value represents a feature value. The coefficient *a*_*i*_ in (9) is to be determined next.

The coefficient determination problem is basically an optimization problem. An unconstrained optimization problem is formed by adding a term, called penalty function, to the objective function. The penalty function consists of a penalty parameter multiplied by a measure of violation of some constraints. In the case of overfitting problem, we consider a penalty function *S*(*a*_*i*_) in terms of penalty coefficient *c*_*i*_:

(10) $$\begin{array}{c} S\left(a_{i}\right)=\lambda \cdot \overset{n}{\underset{i=1}{\sum }}c_{i}\cdot a_{i}^{2}, \end{array}$$

where *λ* is a penalty parameter. Similar to solving a normal regression problem, we look for each calculated feature *f*(*h*_*k*_, *a*_*i*_) that could be as close to the measurement value *x*(*k*) as possible. Do note that *x*(*k*) is the value after using the proposed KF. The final penalty regression curve is computed by minimizing the cost function *E* as follows:

(11) $$\begin{array}{c} E=\alpha \cdot \overset{M}{\underset{k=1}{\sum }}{\left(f\left(h_{k},a_{i}\right)-x\left(k\right)\right)}^{2}+S\left(a_{i}\right), \end{array}$$

where *α* is called learning rate. To find the minimum value of the cost function for the *M* samples (after applying the proposed KF) in terms of the coefficient *a*_*i*_, we use the well-known gradient descend algorithm comprising of the following two main equations:

(12) $$\begin{array}{c} \frac{\partial E}{\partial a_{0}}=2\alpha \cdot \overset{M}{\underset{k=1}{\sum }}\left(f\left(h_{k},a_{i}\right)-x\left(k\right)\right)=0, \end{array}$$

(13) $$\begin{array}{c} \frac{\partial E}{\partial a_{i}}=2\alpha \cdot \overset{M}{\underset{k=1}{\sum }}\left(f\left(h_{k},a_{i}\right)-x\left(k\right)\right)\cdot h_{k}^{i}+2\lambda \cdot c_{i}\cdot a_{i}=0. \end{array}$$

By assigning appropriate values for the coefficient *c*_*i*_, we can inhibit the increase for a particular higher-order coefficient *a*_*i*_ to avoid overfitting.

Since there is no closed-form solution for *a*_*i*_′*s* based on (12) and (13), an iterative computer procedure is generally required. We use an existing MATLAB function for this problem. When we get all the *a*_*i*_′*s*, the relationship between Hb level *h*_*k*_ and the feature value *f*(*h*_*k*_, *a*_*i*_) is established. Once we have extracted the feature value from a new sample image, the corresponding Hb level would be estimated immediately according to this relationship.

### 2.4. Evaluation Schemes

Here, we proposed a three-level evaluation scheme to examine the performance of the anemia detection methods based on how risky a patient may have anemia. Therefore, it is called risk evaluation scheme (RES). The RES extends the traditional evaluation approach that classifies a sample in two levels, one for anemia and another for nonanemia. The RES considers the uncertainty or possible classification error for the samples located around the borderline separating anemia and nonanemia and adding one more level for those samples. Specifically, the RES is designed to separate the samples into three different levels, which are high risk, doubtful, and low risk in terms of the chance of having anemia. In RES, we consider an error tolerance range (ETR) for each individual sample because the KF cannot eliminate all the errors completely, especially the variation from individual differences.

**Figure 4:**
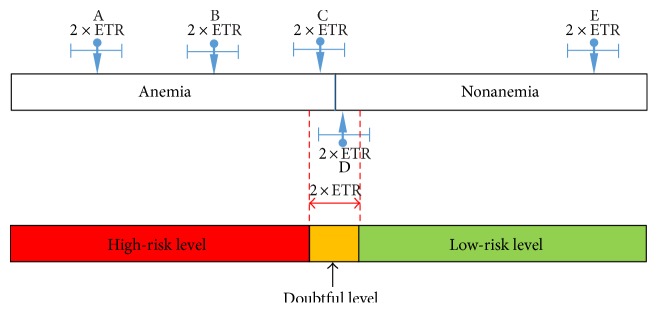
Illustration of RES.

The ETR is based on the idea that each sample cannot stay in one position, but within a certain range. Similarly, the threshold borderline for anemia and nonanemia separation cannot not stay at a fixed location either.  Figure 4 shows two parallel horizontal Hb level bars. In the top bar, an anemic threshold indicated by a vertical line segment is given and the threshold could be 11 g/dL or other choices. Each arrow points to a sample value, say *x*, and the corresponding horizontal line segment containing the arrow represents an interval of length 2 × ETR. To be more specific, the interval containing *x* is from *x* − ETR to *x* + ETR. When *x* happens to be the threshold (say, *x* = 11 g/dL), the corresponding interval defines the doubtful level as indicated in the yellow region of the bottom bar. Similarly, the Hb range from zero to the left boundary of the doubtful interval is defined as the interval having a high-risk level (or the red area in the bottom bar), and the remaining Hb range is defined as the interval having a low-risk level (or the green area in the bottom bar). For the samples whose intervals touch the interval of doubtful levels such as C and D, they have higher chances of being misclassified to the opposite status, that is, from anemia to nonanemia and vice versa. These samples are called suspect samples. The remaining samples are therefore called nonsuspect samples, and they are either in the high-risk level or in the low-risk level. For example, in Figure 4, A and B are in the high-risk level, while E is in the low-risk level.

Since the intervals determined here are based on the choice of ETR and a particular set of training data, they can vary from different training sets even when we set a fixed ETR. In the experiment of Section 3, the ETR is chosen to be the standard deviation of the training data, making the determination of interval depend more on training data. To evaluate the anemia detection methods using testing data, we define an index for each risk level as follows:

(14) High‐risk index=no. of labeled anemic samples in high‐risk leveltotal no. of samples in high‐risk level,

(15) $$\begin{array}{c} Low‐risk\;index=\frac{no.\;of\;labeled\;nonanemic\;samples\;in\;low‐risk\;level}{total\;no.\;of\;samples\;in\;low‐risk\;level}, \end{array}$$

(16) $$\begin{array}{c} Doubtful\;index=\frac{total\;no.\;of\;nonsuspect\;samples}{total\;no.\;of\;samples}. \end{array}$$

Since the indices are evaluated in terms of testing data, while the corresponding risk levels are determined by training data, the performance evaluation of the anemia detection methods can be more reliable in this way. The possible values of each index are all in (0, 1) and higher values are preferred. The first two indices indicate how reliable the anemia detection methods can determine anemia and nonanemia cases, respectively, with high confidence. The doubtful index evaluates the screening capability of the methods to differentiate anemia and nonanemia cases. Samples that are predicted to be in the doubtful level (i.e., suspect samples) are more likely to be uncertain. More suspect samples imply poorer screening capability of the anemia detection methods because further examination, including real blood test, is required for the uncertain cases. The doubtful index evaluates the capability of the anemia detection methods to push suspect samples away into the high-risk level or low-risk level. Obviously, an excellent algorithm would have less estimated samples in the doubtful level (contributing to a higher value of the doubtful index), higher value of the high-risk index, and higher value of the low-risk index. Note that the doubtful interval is adjustable in practice by controlling the value of ETR. For example, if a physician requires less doubt or more confidence in anemia diagnosis, the interval can be extended so that more data will fall in this interval and less data will be considered as high risk or low risk. Further discussion and numerical values of these indices are given in Section 3.2.

## 3. Experimental Results and Discussion

In our experiment, we use the same palpebral conjunctiva images in [13, 14] as our database. There are a total of 100 images in which 40 of them are labeled as anemia samples and 60 of them are labeled as nonanemia samples according to the threshold set at 11 g/dL [13, 14]. In other words, those with an Hb level higher than 11 g/dL are labeled as anemia patients in this paper; otherwise, they would be nonanemic patients. All cases are adult patients and the Hb levels of all of them are examined prior to the treatment. Given the database, we use 10-fold cross validation to evaluate the performance of the proposed approach. In other words, 90 images are used for training and 10 images are used for testing each time and the overall performance is the average result of 10 times (i.e., a total of 100 test data are averaged).

In our previous works ([13, 14]), we enlarged the images to 500 × 500 pixels in order to observe the image texture better and facilitate the further processing of images (e.g., extract entropy features). The two articles ([13, 14]) use the same database, where the original images are usually not square and their sizes vary significantly, but all of them are less than 150 × 150 pixels. All available images are resized (enlarged) to a fixed size of 500 × 500 pixels using relevant MATLAB functions. To comply with those two works, we also do the same thing here. The resulting images indeed become finer in this way and the image characteristics is more obvious.

To initialize the first estimate value in our modified KF, let *x*(1) = *z*(1). For the penalty regression, we let *n* = 4. If we choose a polynomial order higher than 4, the final regression curve stays almost unchanged at the price of additional computing cost. Furthermore, we attempt to avoid the overfitting problem by punishing the higher-order term and inhibiting the growth of its coefficient. The key of choosing penalty parameters *c*_1_–*c*_4_ and *λ* is to reduce the contribution from higher-order terms (to avoid overfitting or overtraining), which reduces the curvature of our fitting line and makes it smoother. So, the assignment principle for the parameters is as follows: The parameter values for lower-order terms are set smaller (lower penalty) and those for higher-order terms are set larger (higher penalty). The fitting algorithm is based on gradient descend, which means it would still be capable of finding the fitting line as long as we follow the assignment principle just mentioned. For example, even if the values for *c*_2_–*c*_4_ are set much greater than *c*_1_, the final fitting line can be obtained (it would look like the result of linear fitting). Thus, the choice of *c*_1_–*c*_4_ is customized by the user subject to the assignment rule just discussed. The parameter *λ* is chosen such that the penalty term would not be too large to make the convergence difficult. In our case, the value assigned to the penalty parameter is smaller than *α* in order not to make the convergence difficult. For *α*, it is the step size in the iteration process. Technologically, we should assign *α* a value that is as small as possible in order not to miss the extremal point in the optimization process. But, the computational cost would be relatively high accordingly. The final results would be the same if *α* is limited to within a range. For example, through our simulation, if the value range for *α* is under 9 × 10^−11^, the final results remain the same generally.

Thus, we set *c*_1_ = 1, *c*_2_ = 10, *c*_3_ = 20, and *c*_4_ = 30 empirically, since we want to keep higher-order coefficients closer to zero. We set a random value from 0 to 1 for the initial value of *a*_*i*_. Considering the performance and the convergence speed, we let *a*_0_ be the first value of the feature data and set learning rate *α* = 1 × 10^−11^ and penalty parameter *λ* = 1 × 10^−12^. The loop termination condition is met when the two objective cost functions in two successive iterations differ by less than 1 × 10^−6^.

### 3.1. Experimental Results of the Proposed Approach

**Figure 5:**
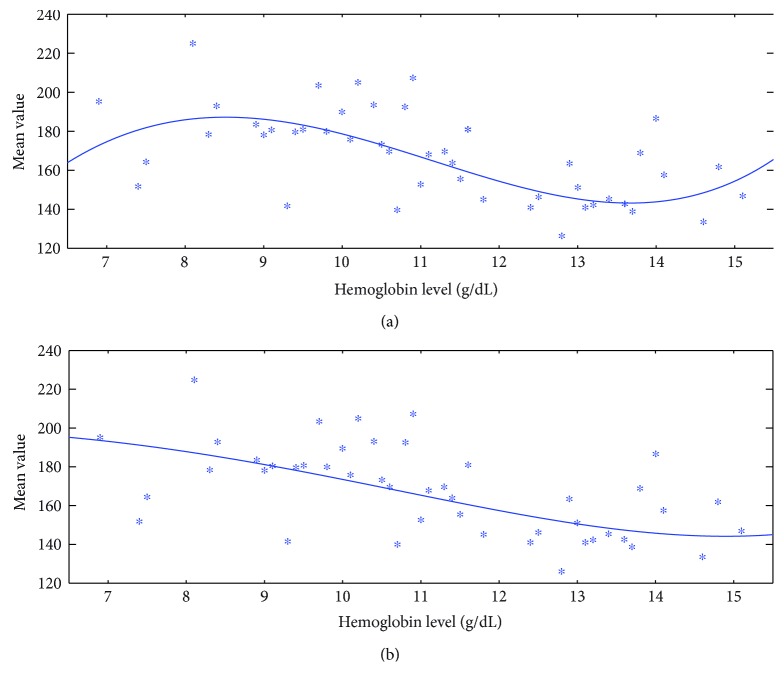
A typical result for the training data without proposed Kalman filtering and the fitting curve (a) without and (b) with penalty function.

**Figure 6:**
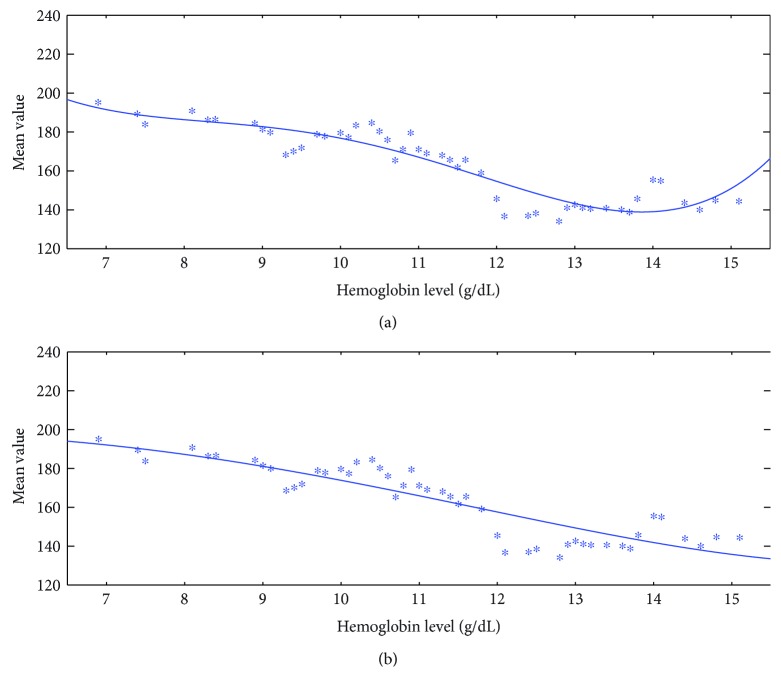
A typical result for the training data with proposed Kalman filtering and the fitting curve (a) without and (b) with penalty function.

Comparing Figures 5() and 6(), we can see that the data (denoted by asterisks) after applying the proposed Kalman filtering has lower data variation, which facilitates the use of regression analysis in the next step. For the fitting curve without penalty function in Figure 5(a), a serious overfitting problem occurs due to great data fluctuation. We can see that the mean value around 180 has roughly two different corresponding Hb levels, which will have a matching issue during the testing phase. In contrast to the regression curve with penalty function in Figure 5(b), it can better present the decreasing trend of the feature. In addition, the regression curve with penalty function is robust that it changes little before and after applying the proposed Kalman filtering. Furthermore, for the no-penalty regression in Figure 6(a), the matching issue still exists even though the data fluctuation is reduced after applying the proposed KF. The fitting curve in Figure 6(b) gets around the problems of both matching issue and great data fluctuation. Therefore, Kalman filtering helps to improve the fitting accuracy and penalty function helps to improve the fitting robustness.

### 3.2. Applying the Proposed Approach to Selected Methods

In addition to our proposed methods, we also examine several related anemia assessment methods using the RES proposed in Section 2.4. The first one includes the use of a linear regression for R color component only (adopted from [38], where it originally considered all R, G, and B components as a whole for regression). The second one uses the erythema index (defined as the logarithm of the R component value) and linear regression [10]. The third one uses the hue color component in the HSI color model as the recognition feature and perform anemia classification manually [39]. However, for the convenience of comparison, we use our nonlinear penalty regression method to replace the manual classification part. These methods are chosen for comparison because they are related to ours as follows: (1) they all use only one simple recognition feature, such as R component value, erythema index, and hue component value, to determine the anemia status, and (2) the first two methods also implement a regression approach for Hb level estimation. None of them includes KF or any other filters in their original methods.

**Figure 7:**
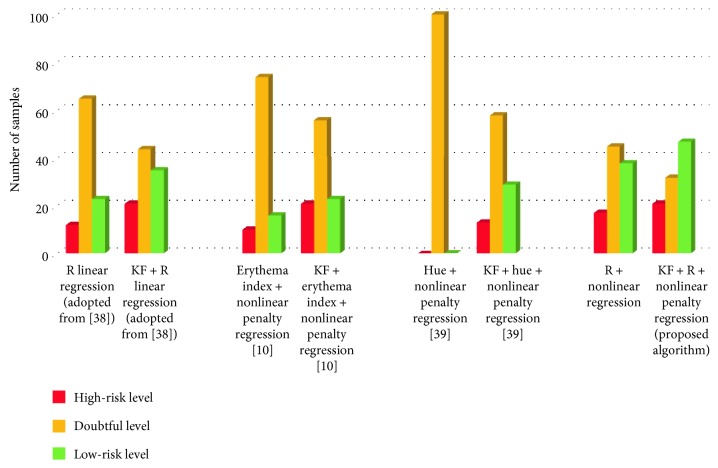
Comparison of the results with and without proposed KF for four different methods.

In the following, we compare the results of all the methods considered before and after applying the proposed KF. The results are shown in Figure 7 and Table 1. Unlike the samples in low- or high-risk levels, anemic samples in the doubtful level do not appear to have obvious color features. The Hb level threshold of 11 g/dL or any specific threshold value is commonly viewed as a “golden” standard to differentiate anemia and nonanemia. However, it is not a “perfect” one. For example, not every patient with 10.9 g/dL can be diagnosed as anemic with great confidence. In a general medical case, a doctor or a researcher may consider a disease in more than one level. The patients with minor symptom or asymptomatic may fall in level 1 and others in another level, say level 2. The treatment plans for the patients in level 1 are more conservative because the doctor is not sure if the patients really have the disease. Back to our case, the samples in the doubtful level are more likely in the uncertain zone. Reducing the number of such samples becomes our primary task. The results of Figure 7 show that all the methods can reduce the number of suspect samples after applying the modified KF. Especially for [39], it can hardly identify any anemic samples or nonanemic samples with confidence before the use of KF, since the data variation is too large for the method. However, it can reduce the number of suspect samples from 100 to 58 after applying KF and regain the capability of identifying anemic samples with confidence.

Table 1 shows the performance comparison using RES, where ETR is selected as the standard deviation of the training data. The sensitivity and specificity criteria follow their well-known definitions except that only the nonsuspect samples are considered. From Table 1, we can see that the sensitivity and specificity performance for some methods have declined slightly after using the proposed KF, but the KF reduces both the standard deviation of feature values and the number of suspect samples. From the aspect of practical medical application, a physician needs to give patients the correct information as much as possible, and thus, an appropriate reduction in the numbers of suspect samples is desirable. Although some methods in Table 1 show very high performance in sensitivity and specificity such as the one in [10], they have too many suspect samples (74 out of 100), which means the corresponding subjects may still need blood test for further verification just in case. Then, the screening purpose of any noninvasive anemia assessment study is hardly met. Therefore, our work is to reduce the occurrence of suspect samples as much as possible, which is equivalent to reducing the number of suspect samples in doubtful level. Although with slight decreasing in sensitivity and specificity, according to the last column, the proposed KF can reduce the number of suspect samples significantly for all the methods considered here.

Of course, we can also change ETR in RES to achieve customized results. The choice of ETR is obviously a trade-off. As discussed in Section 2.4, a larger range would produce more uncertain (suspected) data, while achieving higher accuracy on certain data and vice versa.  Table 2 shows the results of different methods (with the proposed KF) using (14), (15), and (16) in RES. Generally speaking, these methods perform quite well among the nonsuspect samples except [39]. Then, the primary task of noninvasive anemia examining becomes reducing as many suspect samples as possible. The method of KF + hue + nonlinear penalty regression [39] has the worst result with the lowest high-risk, low-risk, and doubtful indices. For the rest of the methods in Table 2, the method of KF + R + linear regression has comparable results with ours. However, it has 0.56 in the doubtful index, which is worse than that of ours. For the method of KF + erythema index + linear regression [10], it has excellent results of 0.9545 and 1.0000 in the high-risk index and low-risk index, respectively, but poor result of 0.44 in doubtful index due to too many suspect samples.

Originally, KF is often used to smooth time-varying data. Now, we apply it to deal with the feature without any time-varying nature, especially in medical applications. It demonstrates how to reduce measurement error. In fact, many medical-related data are time irrelevant, which means our work has the potential use in many other medical applications. Since no research works discuss how a KF can be applied to a time-unrelated medical field to the best of our knowledge, one contribution of our work is to present a new extending application of KF. With the proposed Kalman filtering, physicians and researchers could operate regression algorithms more effectively. Thus, KF may become a useful option for medical applications. Another contribution of our work is to propose a three-level evaluation scheme for anemia detection methods, which is particularly useful if anemia screening is the main objective of the methods.

## 4. Conclusion

In this paper, we proposed a combining approach consisting of the modified Kalman filtering and penalty regression for noninvasive anemia detection. The KF is reformulated so that it can be applied to time-irrelevant medical data. With our proposed KF, the variance of the data could be reduced which can facilitate the pattern-recognition task at a later stage. The penalty regression reduces the chance of overfitting, resulting in a more robust prediction system. We also propose a risk evaluation scheme and the idea of suspect samples. The suspect samples are those difficult to determine for sure whether we have an anemia case or not. The experimental results show that the proposed KF can effectively reduce the number of suspect samples for our proposed method and other relevant methods. This is a great advantage for the screening purpose of noninvasive anemia detection methods. We have proposed a simple and easy-to-use anemia detection method, which can be transformed into wearable or mobile devices if required. In the future, we would explore the use of the proposed KF for other medical applications.

## Figures and Tables

**Table 1:** Performance comparison between the methods with and without KF.

	Sensitivity	Specificity	Number of suspect samples	Sensitivity change	Specificity change	Number of suspect samples change
R + linear regression (adopted from [38])	0.8333	0.8261	65	+2.86%	−6.62%	**−32.31%**
KF + R + linear regression (adopted from [38])	0.8571	0.7714	**44**			
Erythema index + linear regression [10]	1.0000	1.0000	74	0%	−4.35%	**−24.34%**
KF + erythema index + linear regression [10]	1.0000	0.9565	**56**			
Hue + nonlinear penalty regression [39]	^∗^NAN	^∗^NAN	100	^∗^NAN	^∗^NAN	**−42%**
KF + hue + nonlinear penalty regression [39]	0.8462	0.5862	**58**			
R + nonlinear penalty regression	0.7647	0.8158	45	−0.36%	−0.89%	**−28.89%**
KF + R + nonlinear penalty regression (proposed algorithm)	0.7619	0.8085	**32**			

^∗^NAN: there is no data, which means all test samples are considered to be suspect samples.

**Table 2:** The index of each level in RES.

	High-risk index	Doubtful index	Low-risk index
KF + R + linear regression (adopted from [38])	0.6923	0.56	0.9000
KF + erythema index + linear regression [10]	**0.9545**	0.44	**1.0000**
KF + hue + nonlinear penalty regression [39]	0.4783	0.42	0.8947
KF + R + nonlinear penalty regression (proposed algorithm)	0.6400	**0.68**	0.8837
